# Comparison of Cancer-Related Spending and Mortality Rates in the US vs 21 High-Income Countries

**DOI:** 10.1001/jamahealthforum.2022.1229

**Published:** 2022-05-27

**Authors:** Ryan D. Chow, Elizabeth H. Bradley, Cary P. Gross

**Affiliations:** 1 MD-PhD Program, Yale School of Medicine, New Haven, Connecticut; 2 Vassar College, Poughkeepsie, New York; 3 Department of Internal Medicine, Yale School of Medicine, New Haven, Connecticut; 4 Yale Cancer Outcomes, Public Policy, and Effectiveness Research (COPPER) Center, New Haven, Connecticut

## Abstract

**Importance:**

Studies using data from before 2011 concluded that the cost of US cancer care is justified given improved outcomes compared with European countries. However, it is unclear whether contemporary US cancer care provides better value than that of other high-income countries.

**Objective:**

To assess whether cancer mortality rates in 2020 were lower in countries with higher cancer-related spending, and to estimate across countries the incremental cost per averted cancer death.

**Design, Setting, and Participants:**

Cross-sectional, national-level analysis of 22 high-income countries, assessing the association between cancer care expenditures and age-standardized population-level cancer mortality rates in 2020, with and without adjustment for smoking. In addition, US incremental costs per averted death compared with the other countries were calculated. This study was conducted from September 1, 2021, to March 31, 2022.

**Main Outcomes and Measures:**

Age-standardized population-level cancer mortality rates.

**Results:**

In this cross-sectional study of 22 countries, the median cancer mortality rate was 91.4 per 100 000 population (IQR, 84.2-101.6). The US cancer mortality rate was higher than that of 6 other countries (86.3 per 100 000). Median per capita spending in USD for cancer care was $296 (IQR, $222-$348), with the US spending more than any other country ($584). After adjusting for smoking, 9 countries had lower cancer care expenditures and lower mortality rates than the US. Of the remaining 12 countries, the US additionally spent more than $5 million per averted death relative to 4 countries, and between $1 and $5 million per averted death relative to 8 countries. Cancer care expenditures were not associated with cancer mortality rates, with or without adjustment for smoking (Pearson *R* = −0.05 [95% CI, −0.46 to 0.38]; *P* = .81; and *R* = −0.05 [95% CI, −0.46 to 0.38]; *P* = .82).

**Conclusions and Relevance:**

In this cross-sectional study of national cancer care expenditures and cancer mortality rates across 22 countries, although the cancer mortality rate in the US was lower than the median, the US spent twice as much on cancer care as the median country. Findings of this study suggest that the US expenditure on cancer care may not be commensurate with improved cancer outcomes.

## Key Points

**Question:**

Is spending on cancer care associated with lower cancer mortality rates?

**Findings:**

In this cross-sectional study of 22 high-income countries, national cancer care expenditures in 2020 were not associated with age-standardized cancer mortality rates. Although the US had the highest per capita spending on cancer care, after adjustment for smoking, the US cancer mortality rate was comparable with that of the median high-income country.

**Meaning:**

Results of this cross-sectional study suggest that understanding how countries outside the US achieve lower cancer mortality rates with lower spending may prove useful to future researchers, clinicians, and policy makers seeking to best serve their populations.

## Teaser

This cross-sectional study assesses whether cancer mortality rates in 2020 were lower in countries with higher cancer-related spending and estimates across countries the incremental cost per averted cancer death.

## Introduction

For decades, the US has spent more per capita on health care than any other country.^1,2,3^ Health care expenditures in the US have almost doubled from $1.9 trillion in 2000 to $3.8 trillion in 2019.^4,5^ Spending on cancer care in the US has followed a similar pattern, surpassing $200 billion in 2020.^6,7^ These expenses impose a substantial burden on patients, with annual out-of-pocket spending on cancer care estimated at $16 billion.^8^ Furthermore, an estimated 12% to 62% of cancer survivors report being in debt because of their treatment.^9^ The substantial US expenditure in cancer care raises the question of its value: are the high expenditures on cancer care in the US accompanied by lower cancer mortality rates, particularly in comparison with other high-income countries?

Previous studies from 2011 to 2015^10,11,12^ have compared US cancer expenditures and outcomes with those of other countries. Much has changed in cancer care since then. A total of 114 novel cancer drugs were approved by the US Food and Drug Administration from 2011 to 2020,^13^ and prices for existing and newly approved cancer drugs in the US have increased by 12% annually from 1995 to 2014.^14,15,16,17^ Mean drug launch prices increased from $22 000 per year in 2000 to $175 000 in 2018,^15,16,18^ and the US regulatory environment facilitates early adoption of new drugs years before they are reimbursed in other countries.^19^ Accordingly, an updated analysis using contemporary data on population-level cancer care expenditures and cancer mortality rates in the US relative to other high-income countries is warranted.

Previous studies have used varying approaches to compare international cancer care spending and outcomes, each with methodologic limitations. One study reported that patients in the US with cancer experienced a greater increase in survival time between 1983 and 1999 compared with patients in Europe, concluding that the survival gain offset the higher growth rate of cancer spending in the US^10^; however, international differences in cancer survival times may be explained in part by screening practices, in which artifactual survival gains are observed in countries with more aggressive screening programs. Recognizing this limitation, another study compared population-level cancer mortality rates and spending in the US with those of Western Europe from 1982 to 2010.^11^ The authors concluded that the US provides lower value care compared with Western Europe across a number of individual cancer types. This analysis, however, relied on the assumption that expenditures were similar for each cancer type—an assumption that is inconsistent with existing evidence.^6,20^

Another gap in the existing literature is that prior studies have not accounted for international variation in smoking,^21^ which is a prominent risk factor for cancer mortality.^22,23,24^ Without adjusting for national smoking rates, countries with lower smoking rates (such as the US) could appear to have better cancer outcomes for reasons that are independent of cancer-related care. In the examination of the association between cancer care spending and cancer mortality rates, national smoking rates are therefore important to consider.

To further our understanding of value in cancer care at the national level, we asked 3 questions: (1) What is the variation in contemporary cancer-related health care spending across high-income countries? (2) What is the association between national cancer-related spending and cancer mortality rates, with and without adjusting for differences in smoking rates? (3) What is the incremental spending associated with each averted death in the US compared with its international counterparts?

## Methods

This study was deemed exempt from review per Yale University policy (45CFR46.101[b][4]) because it involved the collection or study of existing data. We followed the Strengthening the Reporting of Observational Studies in Epidemiology (STROBE) reporting guideline for cross-sectional studies.

### Study Design and Sampling

We conducted a cross-sectional analysis of cancer care expenditures and age-standardized population-level mortality rates among 22 of the 34 high-income countries in the Organisation for Economic Co-operation and Development (OECD)^25^ in 2020. Countries were included if they met 4 criteria: (1) having a very high Human Development Index, a measure of a country’s status in several dimensions of human development, as determined by the United Nations^26^; (2) being in the top 30 countries based on gross national income per capita, which was selected based on sample sizes used in prior studies^10,11^ and a power calculation for correlation (assuming α = .05, β = 0.20, and *R* = 0.5); (3) having 2020 cancer mortality rate estimates; and (4) having recent estimates (after 2010) for the percentage of health care expenditures dedicated to cancer care. Our primary outcome was age-standardized cancer mortality rates. This study was conducted from September 1, 2021, to March 31, 2022.

### Data Sources and Measures

We estimated per capita cancer care spending in each country in 2019 using OECD data on total health expenditures and the percentage of health expenditures on cancer care. All monetary values are expressed as values in 2021 US dollars. All data used in this study were publicly available. From the GLOBOCAN database, we extracted age-standardized cancer mortality rate estimates in 2020 for the 22 selected countries, including all 36 cancers represented in the database.^27^ Using the OECD database,^25^ we obtained 2019 estimates for total health expenditures. We also compared the OECD database to the Global Health Expenditure Database and found nearly identical expenditure estimates. We gathered country-specific estimates of the percentage of health expenditures dedicated to cancer care from the most recent available data.^6,28,29,30,31,32,33^ Due to limited data availability, these percentages were derived from spending data in years ranging from 2012 to 2018, with the most frequent reference year being 2018 (18 of 22 countries, 82%). We used the World Bank database^34^ to extract population data in 2019 for each country. Finally, we collected data on smoking rates in 1996 for each country as determined from major multinational surveys,^21^ given the 20- to 30-year lag between changes in smoking rates and population-level cancer mortality rates^35,36^ (eTable in the Supplement).

We considered whether countries with lower noncancer disease burden may experience higher cancer mortality rates. We obtained life expectancy projections at age 60 from the United Nations World Populations Prospect report.^37^ This metric was chosen because the mean age of cancer diagnosis is approximately 60 years^38^ and would thus offer the most direct comparison with cancer mortality rates.

### Statistical Analyses

Our primary analysis investigated the association between cancer care spending and cancer mortality rates across countries using linear regression and correlation. We additionally adjusted for smoking rates to assess the association between cancer care spending and smoking-adjusted cancer mortality rates. As a sensitivity analysis, we adjusted cancer care expenditures by economywide purchasing power parity, an approach to adjust for differences in the cost of living across countries. In our secondary analysis, we compared each country with those with the highest cancer mortality rate or highest cancer care expenditures, calculating the ratio of additional spending and averted deaths in each country.

To determine per capita cancer care spending for each country, we converted 2019 total health expenditures to USD using 2019 exchange rates.^39^ We multiplied total health expenditures by the percentage that was spent on cancer care. We then divided 2019 cancer care spending by the total 2019 population in each country to obtain per capita cancer expenditures.

For our primary analysis, we performed linear regression with cancer care expenditures as the independent variable and age-standardized cancer mortality rates as the dependent variable, with and without adjustment for smoking. We calculated the predicted mortality rate after accounting for smoking (smoking-adjusted mortality) by summing the intercept and residuals from a linear regression model of cancer mortality rates as a function of smoking. We also calculated Pearson correlation coefficients between cancer expenditures and cancer mortality rates, or with smoking-adjusted mortality rates. As a sensitivity analysis, we assessed purchasing power parity–adjusted cancer expenditures and mortality rates. All statistical analyses were performed in R version 4.0.2 (R Foundation for Statistical Computing). Statistical significance was defined as 2-sided *P* < .05.

For our secondary analysis, we used the country with the highest spending (the US) during the study period as the reference. Comparative analyses were performed using smoking-adjusted mortality rates. Comparing cancer mortality rates and expenditures relative to the US, we subtracted the smoking-adjusted mortality rate (per 100 000 population) in the US from that of each comparator country to obtain the number of cancer deaths averted (per 100 000 population). We then subtracted cancer care expenditures (per 100 000 population) in each country from that of the US to calculate the incremental cost from the US perspective. In addition, we divided incremental cancer care spending by the number of cancer deaths averted to determine incremental spending per averted cancer death. We categorized each of the 21 comparator countries into 3 groups: (1) countries with lower costs and mortality rates than the US, (2) countries in which the US spent $1 to $5 million per averted death, and (3) countries in which the US spent more than $5 million per averted death. These thresholds were selected based on the observed distribution of incremental costs per death averted. Similarly, we took the country with the highest cancer mortality rate (Denmark) as the reference, comparing Denmark with countries with higher expenditures but lower cancer mortality rates.

## Results

Our final sample included 22 high-income countries. The median age-standardized cancer mortality rate per 100 000 standard population was 91.4 (IQR, 84.2-101.6), with Denmark having the highest mortality rate at 113.7 deaths per 100 000 (Table). The US cancer mortality rate was higher than that of 6 other countries, at 86.3 deaths per 100 000. Korea had the lowest cancer mortality rate, at 75.5 deaths per 100 000. Consistent with cancer being a major cause of death in the elderly population,^40^ life expectancy at age 60 was negatively correlated with cancer mortality rates (Pearson *R* = −0.49 [95% CI, −0.75 to −0.08]; *P* = .02) (eFigure 1 in the Supplement). Cancer care accounted for a median of 6.0% (IQR, 4.9%-6.9%) of total health care spending (Table). The percentage of health care spending dedicated to cancer care was lowest in Sweden (3.7%) and highest in Korea (9.6%). The median per capita spending for cancer care was $296 (IQR, $222-$348), ranging from $132 in Spain to $584 in the US.

**Table.** Characteristics of Included Countries

Country	Age-standardized cancer mortality per 100 000	Smoking-adjusted cancer mortality per 100 000	Total health costs per capita, $^a^	Cancer care (% of total health costs)	Reference year for the % of health spending on cancer care	Cancer care costs per capita, $^a^	Life expectancy in y at age 60 y
Australia^31^	83.3	64.0	5123	5.93	2015	304	25.7
Austria^29^	95.8	72.1	5231	6.40	2018	335	23.9
Belgium^29^	101.3	75.0	4947	6.90	2018	341	24.2
Canada^28^	93.5	72.7	5023	5.38	2012	270	25.0
Denmark^29^	113.7	85.7	5995	4.80	2018	288	23.4
Finland^29^	84.3	65.8	4463	4.00	2018	179	24.5
France^29^	107.9	80.8	4488	7.10	2018	319	25.6
Germany^29^	102.3	79.9	5437	6.80	2018	370	24.0
Iceland^29^	84	61.2	5900	3.80	2018	224	24.7
Ireland^29^	104.9	80.2	5397	5.00	2018	270	24.6
Italy^29^	91.1	69.7	2911	6.70	2018	195	25.4
Japan^30^	81.5	55.8	4427	7.50	2018	332	26.7
Korea^33^	75.5	50.1	2600	9.60	2018	250	25.1
Luxembourg^29^	87.7	63.6	6161	6.90	2018	425	24.4
Netherlands^29^	107.7	85.6	5318	6.90	2018	367	24.3
New Zealand^32^	99	78.5	3877	6.00	2014	233	24.9
Norway^29^	91.7	66.1	7978	4.20	2018	335	24.5
Spain^29^	90.3	62.9	2701	4.90	2018	132	25.6
Sweden^29^	87.2	69.4	5645	3.70	2018	209	24.7
Switzerland^29^	83.3	57.4	9629	6.00	2018	578	25.7
UK^29^	100.5	76.5	4303	5.00	2018	215	24.0
US^6^	86.3	69.0	10 945	5.33	2015	584	23.6

^a^ All currency values are in 2021 USD.

**Figure 1.**
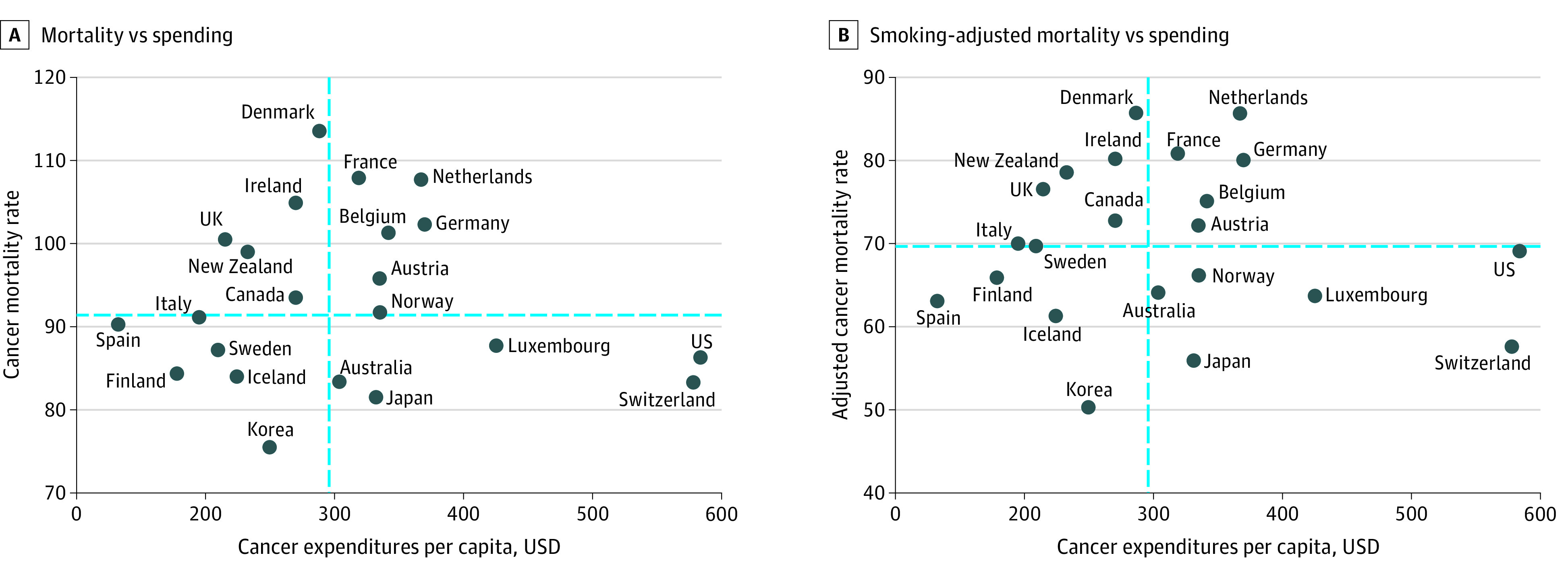
Association Between National Cancer Expenditures and Cancer Mortality Cancer care expenditures in relation to age-standardized cancer mortality rates (deaths per 100 000 standard population). Dashed lines indicate the median. A, Unadjusted cancer mortality compared with cancer care expenditures. B, Cancer mortality compared with cancer care expenditures, after adjustment for smoking rates.

**Figure 2.**
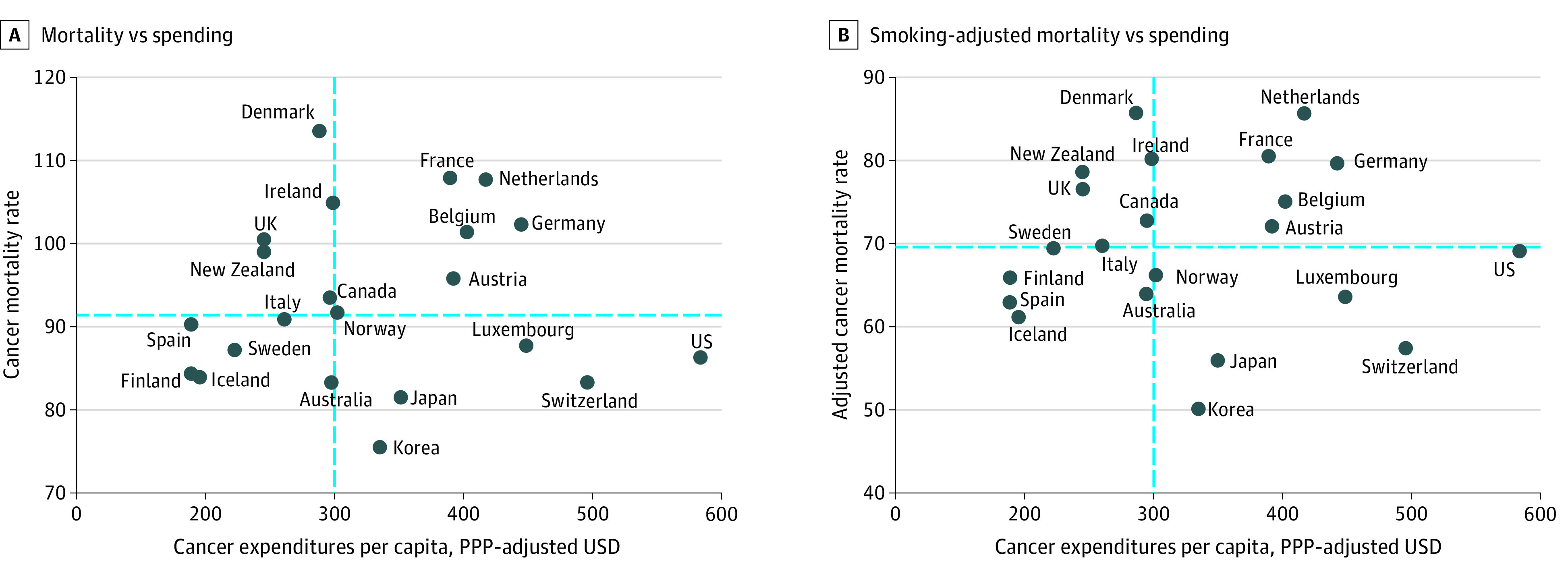
Association Between National Cancer Expenditures Adjusted for PPP (Purchasing Power Parity) and Cancer Mortality Cancer care expenditures adjusted for economywide PPP in relation to total cancer mortality rates (deaths per 100 000 standard population). Dashed lines indicate the median. A, Unadjusted cancer mortality compared with PPP-adjusted cancer care expenditures. B, Cancer mortality compared with PPP-adjusted cancer care expenditures, after adjustment for smoking rates.

After adjusting the analysis for international variations in smoking, the US cancer mortality rate was higher than that of 9 other countries (Figure 1). In the linear regression model, age-standardized cancer mortality rates were not associated with cancer care spending, with adjustment for smoking (multiple *R*^2^ = 0.003, *P* = .81) and without adjustment for smoking (*R*^2^ = 0.003, *P* = .82) (Figure 1). Similarly, cancer care expenditures were not significantly correlated with smoking-adjusted cancer mortality rates (Pearson *R* = −0.05 [95% CI, –0.46 to 0.38]; *P* = .81) or unadjusted cancer mortality rates (Pearson *R* = –0.05 [95% CI, −0.46 to 0.38]; *P* = .82). In a sensitivity analysis of cancer care expenditures adjusted for economywide purchasing power parity to account for cross-national differences in purchasing power, results did not differ substantially (Figure 2); cancer care spending was not significantly correlated with smoking-adjusted cancer mortality rates (Pearson *R* = 0.05 [95% CI, –0.38 to 0.46]; *P* = .84) or unadjusted cancer mortality rates (Pearson *R* = 0.06 [95% CI, –0.37 to 0.47]; *P* = .80).

**Figure 3.**
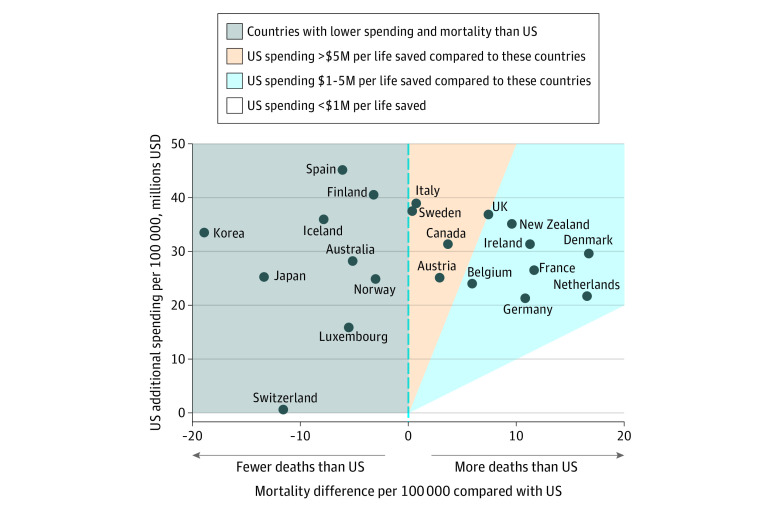
US Incremental Cancer Expenditures per Death Averted Compared With 21 High-Income Countries Incremental spending per cancer death averted, based on age-standardized, smoking-adjusted mortality relative to the US. Additional spending per 100 000 is expressed in millions USD from the US perspective, where positive values indicate increased spending in the US relative to the comparator country. Cancer deaths averted are expressed relative to the US, where positive values indicate more deaths in the comparator country compared with the US. Countries in the gray region had lower spending and mortality than the US. Compared with countries in the blue region, the US spent between $1 and $5 million per averted death. Compared with countries in the orange region, the US spent more than $5 million per averted death.

Relative to the US, the country with the highest per capita spending on cancer care, 9 countries had both lower smoking-adjusted cancer mortality rates and lower spending: Australia, Finland, Iceland, Japan, Korea, Luxembourg, Norway, Spain, and Switzerland (Figure 3). Compared with countries with higher cancer mortality rates than the US, the US additionally spent more than $1 million for each averted cancer death relative to all 12 comparator countries (Figure 3). Of these, the US spent more than $5 million per averted cancer death compared with 4 countries: Sweden (with the US spending an additional $89.3 million for each death averted), Italy ($54.2 million), Canada ($8.5 million), and Austria ($8.0 million).

Compared with Denmark, the country with the highest cancer mortality rate, 10 countries had both lower smoking-adjusted cancer mortality rates and lower cancer expenditures. For the 11 countries that had higher cancer spending than Denmark, we calculated their incremental spending per averted cancer death; the Netherlands (an additional $103.5 million per death averted), the US ($1.8 million), and Germany ($1.4 million) had the highest incremental spending per averted cancer death compared with Denmark (eFigure 2 in the Supplement). Smoking-adjusted cancer mortality rates in Denmark and the Netherlands were similar (85.7 and 85.6 deaths per 100 000, respectively) and expenditures in the Netherlands were 27% higher, resulting in a large ratio of incremental spending to averted deaths (Table).

## Discussion

In this cross-sectional study using contemporary data on cancer mortality rates and cancer care expenditures across 22 high-income countries, we found that national cancer care expenditures were not associated with age-standardized cancer mortality rates. While the US cancer mortality rate was lower than the median within our sample, the US had the highest per capita spending on cancer care. Notably, several countries had both lower expenditures and lower cancer mortality rates than the US. Compared with the remaining countries with higher cancer mortality rates, the US additionally spent more than $1 million per averted cancer death.

Our research builds on prior studies. By conducting country-level comparisons, we were able to adjust for differences in smoking rates across countries. Previous studies either aggregated European countries^10,11^ or categorized countries into spending groups.^12^ Such aggregation may have masked differences in cancer expenditures and outcomes among individual countries. We observed wide variation in the relative cost efficiency of cancer care across European countries, with Germany and the Netherlands providing lower value care than Finland and Iceland. As we report, adjusting for differences in smoking rates shows that US cancer care had less favorable outcomes than suggested by unadjusted mortality rates, as historically lower smoking rates in the US had been protective against cancer mortality.

Our study drew from data from 2019 to 2020, reflecting the continual growth in the costs of novel cancer therapies approved in the past decade. In addition, we focused on age-standardized cancer mortality rates to mitigate issues with overdiagnosis and lead-time bias from international variations in cancer screening approaches. We further normalized national cancer expenditures by the total population rather than incident cancer cases to measure all cancer prevention and treatment programs at the population level.

To place our results in context, the factors associated with the high cost of US cancer care must be considered. Cancer drug expenditures account for 37% of privately insured US cancer expenditures,^41^ and US cancer drug costs are greater than those of other countries.^42^ Prices for the same medications are higher in the US,^16,43,44^ and cancer drugs frequently increase in price after their initial launch.^16,17,45^ This phenomenon stems from the inability of Medicare to negotiate pricing, along with state laws mandating insurers to cover all approved cancer drugs regardless of cost.^19,46^ In addition, the US regulatory environment facilitates earlier and wider access to new drugs compared with other countries.^19,47,48,49^ The US Food and Drug Administration does not consider pricing when evaluating a drug and increasingly grants drug approvals on the basis of preliminary evidence, including unvalidated surrogate measures.^50,51,52,53^ Accordingly, much of the growth in US cancer drug spending has been attributed to monoclonal antibodies, kinase inhibitors, and immune checkpoint inhibitors,^42,54^ which often confer marginal or unclear survival gains.^55,56,57^ Many US Food and Drug Administration–approved cancer drugs are subsequently denied authorization or coverage in England because of deficiencies in safety, efficacy, or cost-effectiveness.^50^

End-of-life care is resource-intensive in the US; within the last 6 months of life, US patients with cancer are admitted to the intensive care unit at twice the rate of other countries and are more likely to receive chemotherapy.^58^ While US cancer screening guidelines are similar to those of other high-income countries,^59^ specialty societies often advocate for more aggressive screening campaigns,^59^ and high rates of inappropriate cancer screening have been reported.^60,61,62,63,64^ Compounding this issue, low-risk tumors such as early-stage prostate cancers are often subject to intervention in the US,^65,66,67^ despite evidence that many of these lesions are unlikely to cause harm if left untreated.^68,69,70^

We also investigated whether countries with lower non-cancer disease burden would have higher cancer mortality rates, as their populations might live long enough to die of cancer rather than other causes. We instead found that countries with higher cancer mortality rates had lower life expectancy at age 60 years, which is consistent with the fact that cancer is a leading cause of death in the elderly.^40^ Of note, the ratio of differential expenditures to averted deaths should be interpreted in the context of absolute rates. We observed that relative to Denmark, the Netherlands spent an additional $103.5 million per death averted, which was much higher than the US ($1.8 million per death averted). This reflects how smoking-adjusted cancer mortality rates in Denmark and the Netherlands were similar and expenditures in the Netherlands were higher, resulting in a large ratio of incremental spending to averted deaths.

### Limitations

This study has limitations. First, our use of total cancer mortality rates may mask international differences in cancer type-specific mortality, a subject that warrants further research examining specific types of cancer. Second, we did not have data on patient-oriented outcomes such as quality of life and the physical, psychological, or financial harms associated with cancer care, although cancer mortality is nonetheless an important outcome. Third, the percentages of health expenditures directed to cancer care were derived from multiple years of data; within a country, however, annual fluctuations in this percentage are limited.^29,71^ Fourth, some countries did not include screening costs as part of cancer-related spending. Because screening costs are negligible relative to overall cancer care expenditures,^31^ their omission or inclusion is unlikely to affect our findings. Fifth, we focused on high-income countries; subsequent studies may explore these questions across a wider cross-section of the world. In addition, we did not incorporate cancer-associated risk factors other than smoking; future work may incorporate other population-level risk factors.

## Conclusions

In this cross-sectional study of 22 high-income countries, cancer care spending was not associated with age-standardized cancer mortality rates. Although the US spent more on cancer care than any other country, this expenditure was not associated with substantially lower cancer mortality rates. Understanding how other countries achieve lower cancer mortality rates at a fraction of US spending may prove useful to future researchers, clinicians, and policy makers seeking to best serve their populations.

## Notes

Supplement. **eTable.** Additional Demographic and Economic Data for the 22 Countries **eFigure 1.** Association Between Life Expectancy After Age 60 and Cancer Mortality **eFigure 2.** Incremental Spending Per Cancer Death Averted Compared With the Highest-Mortality Country (Denmark)

## References

[1] McCullough JM, Speer M, Magnan S, Fielding JE, Kindig D, Teutsch SM. Reduction in US health care spending required to meet the Institute of Medicine’s 2030 target. Am J Public Health. 2020;110(12):1735-1740. doi:10.2105/AJPH.2020.305793 33058710 PMC7661993

[2] Sisko AM, Keehan SP, Poisal JA, . National health expenditure projections, 2018-27: economic and demographic trends drive spending and enrollment growth. Health Aff (Millwood). 2019;38(3):491-501. doi:10.1377/hlthaff.2018.05499 30785832

[3] Papanicolas I, Woskie LR, Jha AK. Health care spending in the United States and other high-income countries. JAMA. 2018;319(10):1024-1039. doi:10.1001/jama.2018.1150 29536101

[4] Martin AB, Hartman M, Lassman D, Catlin A; National Health Expenditure Accounts Team. National health care spending in 2019: steady growth for the fourth consecutive year. Health Aff (Millwood). 2021;40(1):14-24. doi:10.1377/hlthaff.2020.02022 33326300

[5] Centers for Medicare & Medicaid Services. National health expenditure data. Accessed October 20, 2021. https://www.cms.gov/Research-Statistics-Data-and-Systems/Statistics-Trends-and-Reports/NationalHealthExpendData

[6] Mariotto AB, Enewold L, Zhao J, Zeruto CA, Yabroff KR. Medical care costs associated with cancer survivorship in the United States. Cancer Epidemiol Biomarkers Prev. 2020;29(7):1304-1312. doi:10.1158/1055-9965.EPI-19-1534 32522832 PMC9514601

[7] Dieleman JL, Cao J, Chapin A, . US health care spending by payer and health condition, 1996-2016. JAMA. 2020;323(9):863-884. doi:10.1001/jama.2020.0734 32125402 PMC7054840

[8] Yabroff KR, Mariotto A, Tangka F, . Annual Report to the Nation on the Status of Cancer, Part 2: Patient Economic Burden Associated With Cancer Care. J Natl Cancer Inst. 2021;113(12):1670-1682. doi:10.1093/jnci/djab192 34698839 PMC9891103

[9] Altice CK, Banegas MP, Tucker-Seeley RD, Yabroff KR. Financial hardships experienced by cancer survivors: a systematic review. J Natl Cancer Inst. 2016;109(2):djw205. doi:10.1093/jnci/djw205 27754926 PMC6075571

[10] Philipson T, Eber M, Lakdawalla DN, Corral M, Conti R, Goldman DP. An analysis of whether higher health care spending in the United States versus Europe is ‘worth it’ in the case of cancer. Health Aff (Millwood). 2012;31(4):667-675. doi:10.1377/hlthaff.2011.1298 22492882 PMC3829769

[11] Soneji S, Yang J. New analysis reexamines the value of cancer care in the United States compared to Western Europe. Health Aff (Millwood). 2015;34(3):390-397. doi:10.1377/hlthaff.2014.0174 25732488 PMC4436656

[12] Stevens W, Philipson TJ, Khan ZM, MacEwan JP, Linthicum MT, Goldman DP. Cancer mortality reductions were greatest among countries where cancer care spending rose the most, 1995-2007. Health Aff (Millwood). 2015;34(4):562-570. doi:10.1377/hlthaff.2014.0634 25847637

[13] Research AA for C. By the Numbers. By the numbers: novel drugs approved by the FDA, 2011-2020. Cancer Discov. 2021;11(5):1001-1001. doi:10.1158/2159-8290.CD-NB2021-0318 33632776

[14] Howard DH, Bach PB, Berndt ER, Conti RM. Pricing in the market for anticancer drugs. J Econ Perspect. 2015;29(1):139-162. doi:10.1257/jep.29.1.139 28441702

[15] Kantarjian H, Patel Y. High cancer drug prices 4 years later-Progress and prospects. Cancer. 2017;123(8):1292-1297. doi:10.1002/cncr.30545 28182263

[16] Vokinger KN, Hwang TJ, Daniore P, . Analysis of launch and postapproval cancer drug pricing, clinical benefit, and policy implications in the US and Europe. JAMA Oncol. 2021;7(9):e212026. doi:10.1001/jamaoncol.2021.2026 34196656 PMC8251654

[17] Gordon N, Stemmer SM, Greenberg D, Goldstein DA. Trajectories of injectable cancer drug costs after launch in the united states. J Clin Oncol. 2018;36(4):319-325. doi:10.1200/JCO.2016.72.2124 29016226

[18] Dusetzina SB. Drug pricing trends for orally administered anticancer medications reimbursed by commercial health plans, 2000-2014. JAMA Oncol. 2016;2(7):960-961. doi:10.1001/jamaoncol.2016.0648 27123993

[19] Huntington SF, Davidoff AJ, Gross CP. Precision medicine in oncology ii: economics of targeted agents and immuno-oncology drugs. J Clin Oncol. 2020;38(4):351-358. doi:10.1200/JCO.19.01573 31804866

[20] Blakely T, Atkinson J, Kvizhinadze G, Wilson N, Davies A, Clarke P. Patterns of cancer care costs in a country with detailed individual data. Med Care. 2015;53(4):302-309. doi:10.1097/MLR.0000000000000330 25749656 PMC4379114

[21] Ng M, Freeman MK, Fleming TD, . Smoking prevalence and cigarette consumption in 187 countries, 1980-2012. JAMA. 2014;311(2):183-192. doi:10.1001/jama.2013.284692 24399557

[22] Shopland DR. Tobacco use and its contribution to early cancer mortality with a special emphasis on cigarette smoking. Environ Health Perspect. 1995;103(suppl 8):131-142. 8741773 10.1289/ehp.95103s8131 PMC1518977

[23] Shopland DR, Eyre HJ, Pechacek TF. Smoking-attributable cancer mortality in 1991: is lung cancer now the leading cause of death among smokers in the United States? J Natl Cancer Inst. 1991;83(16):1142-1148. doi:10.1093/jnci/83.16.1142 1886147

[24] Islami F, Goding Sauer A, Miller KD, . Proportion and number of cancer cases and deaths attributable to potentially modifiable risk factors in the United States. CA Cancer J Clin. 2018;68(1):31-54. doi:10.3322/caac.21440 29160902

[25] Organisation for Economic Cooperation and Development. Health at a Glance 2019: OECD Indicators. OECD; 2019.

[26] Human Development Report 2020 | UNDP HDR. Human Development Report 2020 | UNDP HDR. Accessed March 27, 2022. http://report.hdr.undp.org

[27] Sung H, Ferlay J, Siegel RL, . Global Cancer Statistics 2020: GLOBOCAN estimates of incidence and mortality worldwide for 36 cancers in 185 countries. CA Cancer J Clin. 2021;71(3):209-249. doi:10.3322/caac.21660 33538338

[28] de Oliveira C, Weir S, Rangrej J, . The economic burden of cancer care in Canada: a population-based cost study. CMAJ Open. 2018;6(1):E1-E10. doi:10.9778/cmajo.20170144 29301745 PMC5878959

[29] Hofmarcher T, Lindgren P, Wilking N, Jönsson B. The cost of cancer in Europe 2018. Eur J Cancer. 2020;129:41-49. doi:10.1016/j.ejca.2020.01.011 32120274

[30] Cancer care and access to cancer drugs in Asia-Pacific - Main report. 2021. IHE. Accessed September 23, 2021. https://ihe.se/en/publicering/cancer-care-and-access-to-cancer-drugs-in-asia-pacific/

[31] Health system expenditure on cancer and other neoplasms in Australia, 2015–16, Summary. Australian Institute of Health and Welfare. Accessed September 9, 2021. https://www.aihw.gov.au/reports/cancer/health-system-expenditure-cancer-other-neoplasms/summary

[32] New Zealand Cancer Plan. Better, faster cancer care 2015–2018. Ministry of Health NZ. Accessed September 23, 2021. https://www.health.govt.nz/publication/new-zealand-cancer-plan-better-faster-cancer-care-2015-2018

[33] Kim JY, Lee KE, Kim K, . Choosing Wisely: The Korean perspective and launch of the ‘Right Decision in Cancer Care’ Initiative. Cancer Res Treat. 2020;52(3):655-660. doi:10.4143/crt.2020.221 32599973 PMC7373865

[34] Population, total | Data. Accessed November 13, 2021. https://data.worldbank.org/indicator/SP.POP.TOTL

[35] Preston SH, Glei DA, Wilmoth JR. A new method for estimating smoking-attributable mortality in high-income countries. Int J Epidemiol. 2010;39(2):430-438. doi:10.1093/ije/dyp360 20032265 PMC2915474

[36] Thun M, Peto R, Boreham J, Lopez AD. Stages of the cigarette epidemic on entering its second century. Tob Control. 2012;21(2):96-101. doi:10.1136/tobaccocontrol-2011-050294 22345230

[37] United Nations, Department of Economic and Social Affairs. World Population Prospects - Population Division - United Nations. 2019. Accessed March 28, 2022. https://population.un.org/wpp/Download/Standard/Mortality/

[38] Weir HK, Thompson TD, Stewart SL, White MC. Cancer incidence projections in the United States between 2015 and 2050. Prev Chronic Dis. 2021;18:E59. doi:10.5888/pcd18.210006 34114543 PMC8220959

[39] Conversion rates - Exchange rates - OECD Data. Accessed November 13, 2021. https://data.oecd.org/conversion/exchange-rates.htm

[40] Gorina Y, Hoyert D, Lentzner H, Goulding M. Trends in causes of death among older persons in the United States. Aging Trends. 2005;(6):1-12. 19174841

[41] Zaorsky NG, Khunsriraksakul C, Acri SL, . Medical service use and charges for cancer care in 2018 for privately insured patients younger than 65 years in the US. JAMA Netw Open. 2021;4(10):e2127784. doi:10.1001/jamanetworkopen.2021.27784 34613403 PMC8495533

[42] Salas-Vega S, Mossialos E. Cancer drugs provide positive value in nine countries, but the United States lags in health gains per dollar spent. Health Aff (Millwood). 2016;35(5):813-823. doi:10.1377/hlthaff.2015.1453 27140987

[43] Goldstein DA, Clark J, Tu Y, . A global comparison of the cost of patented cancer drugs in relation to global differences in wealth. Oncotarget. 2017;8(42):71548-71555. doi:10.18632/oncotarget.17742 29069727 PMC5641070

[44] Salas-Vega S, Shearer E, Mossialos E. Relationship between costs and clinical benefits of new cancer medicines in Australia, France, the UK, and the US. Soc Sci Med. 2020;258:113042. doi:10.1016/j.socscimed.2020.113042 32480184

[45] Shih YT, Xu Y, Liu L, Smieliauskas F. Rising prices of targeted oral anticancer medications and associated financial burden on Medicare beneficiaries. J Clin Oncol. 2017;35(22):2482-2489. doi:10.1200/JCO.2017.72.3742 28471711 PMC5536165

[46] Cherla A, Renwick M, Jha A, Mossialos E. Cost-effectiveness of cancer drugs: comparative analysis of the United States and England. EClinicalMedicine. 2020;29-30:100625. doi:10.1016/j.eclinm.2020.100625 33437948 PMC7788430

[47] Lythgoe M, Krell J, Warner JL, Desai A, Khaki AR. Time intervals between US Food and Drug Administration (FDA) and European Medicines Agency (EMA) new cancer therapy approvals. J Clin Oncol. 2021;39(15)(suppl):1575. doi:10.1200/JCO.2021.39.15_suppl.1575 33600210

[48] Roberts SA, Allen JD, Sigal EV. Despite criticism of the FDA review process, new cancer drugs reach patients sooner in the United States than in Europe. Health Aff (Millwood). 2011;30(7):1375-1381. doi:10.1377/hlthaff.2011.0231 21680577

[49] Samuel N, Verma S. Cross-comparison of cancer drug approvals at three international regulatory agencies. Curr Oncol. 2016;23(5):e454-e460. doi:10.3747/co.23.2803 27803605 PMC5081017

[50] Cherla A, Naci H, Kesselheim AS, Gyawali B, Mossialos E. Assessment of coverage in England of cancer drugs qualifying for US Food and Drug Administration accelerated approval. JAMA Intern Med. 2021;181(4):490-498. doi:10.1001/jamainternmed.2020.8441 33616607 PMC7900925

[51] Del Paggio JC, Berry JS, Hopman WM, . Evolution of the randomized clinical trial in the era of precision oncology. JAMA Oncol. 2021;7(5):728-734. doi:10.1001/jamaoncol.2021.0379 33764385 PMC7995135

[52] Prasad V, Kim C, Burotto M, Vandross A. The strength of association between surrogate end points and survival in oncology: a systematic review of trial-level meta-analyses. JAMA Intern Med. 2015;175(8):1389-1398. doi:10.1001/jamainternmed.2015.2829 26098871

[53] Gyawali B, Hey SP, Kesselheim AS. Assessment of the clinical benefit of cancer drugs receiving accelerated approval. JAMA Intern Med. 2019;179(7):906-913. doi:10.1001/jamainternmed.2019.0462 31135808 PMC6547118

[54] Paliouras S, Pearson A, Barkalow F. The most successful oncology drug portfolios of the past decade. Nat Rev Drug Discov. 2021;20(11):811-812. doi:10.1038/d41573-021-00022-w 33536658

[55] Haslam A, Prasad V. Estimation of the percentage of US patients with cancer who are eligible for and respond to checkpoint inhibitor immunotherapy drugs. JAMA Netw Open. 2019;2(5):e192535-e192535. doi:10.1001/jamanetworkopen.2019.2535 31050774 PMC6503493

[56] Marquart J, Chen EY, Prasad V. Estimation of the percentage of US patients with cancer who benefit from genome-driven oncology. JAMA Oncol. 2018;4(8):1093-1098. doi:10.1001/jamaoncol.2018.1660 29710180 PMC6143048

[57] Fojo T, Mailankody S, Lo A. Unintended consequences of expensive cancer therapeutics—the pursuit of marginal indications and a me-too mentality that stifles innovation and creativity: the John Conley Lecture. JAMA Otolaryngol Head Neck Surg. 2014;140(12):1225-1236. doi:10.1001/jamaoto.2014.1570 25068501

[58] Bekelman JE, Halpern SD, Blankart CR, ; International Consortium for End-of-Life Research (ICELR). Comparison of site of death, health care utilization, and hospital expenditures for patients dying with cancer in 7 developed countries. JAMA. 2016;315(3):272-283. doi:10.1001/jama.2015.18603 26784775

[59] Ebell MH, Thai TN, Royalty KJ. Cancer screening recommendations: an international comparison of high income countries. Public Health Rev. 2018;39(1):7. doi:10.1186/s40985-018-0080-0 29507820 PMC5833039

[60] Brownlee S, Chalkidou K, Doust J, . Evidence for overuse of medical services around the world. Lancet. 2017;390(10090):156-168. doi:10.1016/S0140-6736(16)32585-5 28077234 PMC5708862

[61] Royce TJ, Hendrix LH, Stokes WA, Allen IM, Chen RC. Cancer screening rates in individuals with different life expectancies. JAMA Intern Med. 2014;174(10):1558-1565. doi:10.1001/jamainternmed.2014.3895 25133746

[62] Tan A, Kuo YF, Goodwin JS. Potential overuse of screening mammography and its association with access to primary care. Med Care. 2014;52(6):490-495. doi:10.1097/MLR.0000000000000115 24828844 PMC4158454

[63] Sirovich BE, Welch HG. Cervical cancer screening among women without a cervix. JAMA. 2004;291(24):2990-2993. doi:10.1001/jama.291.24.2990 15213211

[64] Goodwin JS, Singh A, Reddy N, Riall TS, Kuo YF. Overuse of screening colonoscopy in the Medicare population. Arch Intern Med. 2011;171(15):1335-1343. doi:10.1001/archinternmed.2011.212 21555653 PMC3856662

[65] Lu-Yao GL, Albertsen PC, Moore DF, . Fifteen-year survival outcomes following primary androgen-deprivation therapy for localized prostate cancer. JAMA Intern Med. 2014;174(9):1460-1467. doi:10.1001/jamainternmed.2014.3028 25023796 PMC5499229

[66] Hoffman KE, Niu J, Shen Y, . Physician variation in management of low-risk prostate cancer: a population-based cohort study. JAMA Intern Med. 2014;174(9):1450-1459. doi:10.1001/jamainternmed.2014.3021 25023650 PMC4372187

[67] Alvarado M, Ozanne E, Esserman L. Overdiagnosis and overtreatment of breast cancer. Am Soc Clin Oncol Educ Book. 2012;(32):e40-e45. doi:10.14694/EdBook_AM.2012.32.301 24451829

[68] Loeb S, Bjurlin MA, Nicholson J, . Overdiagnosis and overtreatment of prostate cancer. Eur Urol. 2014;65(6):1046-1055. doi:10.1016/j.eururo.2013.12.062 24439788 PMC4113338

[69] Esserman LJ, Thompson IM Jr, Reid B. Overdiagnosis and overtreatment in cancer: an opportunity for improvement. JAMA. 2013;310(8):797-798. doi:10.1001/jama.2013.108415 23896967

[70] Esserman LJ, Thompson IM, Reid B, . Addressing overdiagnosis and overtreatment in cancer: a prescription for change. Lancet Oncol. 2014;15(6):e234-e242. doi:10.1016/S1470-2045(13)70598-9 24807866 PMC4322920

[71] Jönsson B, Hofmarcher T, Lindgren P, Wilking N. The cost and burden of cancer in the European Union 1995-2014. Eur J Cancer. 2016;66:162-170. doi:10.1016/j.ejca.2016.06.022 27589247

