# Supplementary material for: Comparison of Cancer-Related Spending and Mortality Rates in the US vs 21 High-Income Countries
Source: JAMA Health Forum. 2022 May 27;3(5):e221229. doi: 10.1001/jamahealthforum.2022.1229 (PMC9142870; doi:10.1001/jamahealthforum.2022.1229)
Supplement: Supplement. — eTable. Additional Demographic and Economic Data for the 22 Countries eFigure 1. Association Between Life Expectancy After Age 60 and Cancer Mortality eFigure 2. Incremental Spending Per Cancer Death Averted Compared With the Highest-Mortality Country (Denmark) [file jamahealthforum-e221229-s001.pdf]

Supplemental Online Content

Chow RD, Bradley EH, Gross CP. Comparison of cancer-related spending and mortality rates in the US vs 21 high-income countries. *JAMA Health Forum.* 2022;3(5):e221229. doi:10.1001/jamahealthforum.2022.1229

eTable. Additional Demographic and Economic Data for the 22 Countries

eFigure 1. Association Between Life Expectancy After Age 60 and Cancer Mortality

eFigure 2. Incremental Spending Per Cancer Death Averted Compared With the Highest-Mortality Country (Denmark)

This supplemental material has been provided by the authors to give readers additional information about their work.

© 2022 Chow RD et al. *JAMA Health Forum*.

eTable 1: Additional demographic and economic data for the 22 countries

Country	Population size (millions)	GDP per capita	Cigarette smoking rate (% in 1996)[a]
Australia	25.4	$53,079	24%
Austria	8.9	$58,650	30%
Belgium	11.5	$54,710	33%
Canada	37.6	$50,666	26%
Denmark	5.8	$60,335	35%
Finland	5.5	$51,557	23%
France	67.2	$49,377	34%
Germany	83.1	$55,891	28%
Iceland	0.4	$60,082	29%
Ireland	4.9	$89,431	31%
Italy	59.7	$44,851	27%
Japan	126.3	$42,230	32%
Korea	51.7	$42,728	32%
Luxembourg	0.6	$120,962	30%
Netherlands	17.3	$59,469	28%
New Zealand	5.0	$44,917	26%
Norway	5.3	$68,345	32%
Spain	47.1	$42,184	34%
Sweden	10.3	$55,069	22%
Switzerland	8.6	$73,115	32%
UK	66.8	$48,542	30%
US	328.3	$65,298	22%

a. Cigarette smoking rates were taken from 1996, given the well-established 20–30-year lag between smoking and cancer mortality.

© 2022 Chow RD et al. *JAMA Health Forum*.

eFigure 1: Association between life expectancy after age 60 and cancer mortality.

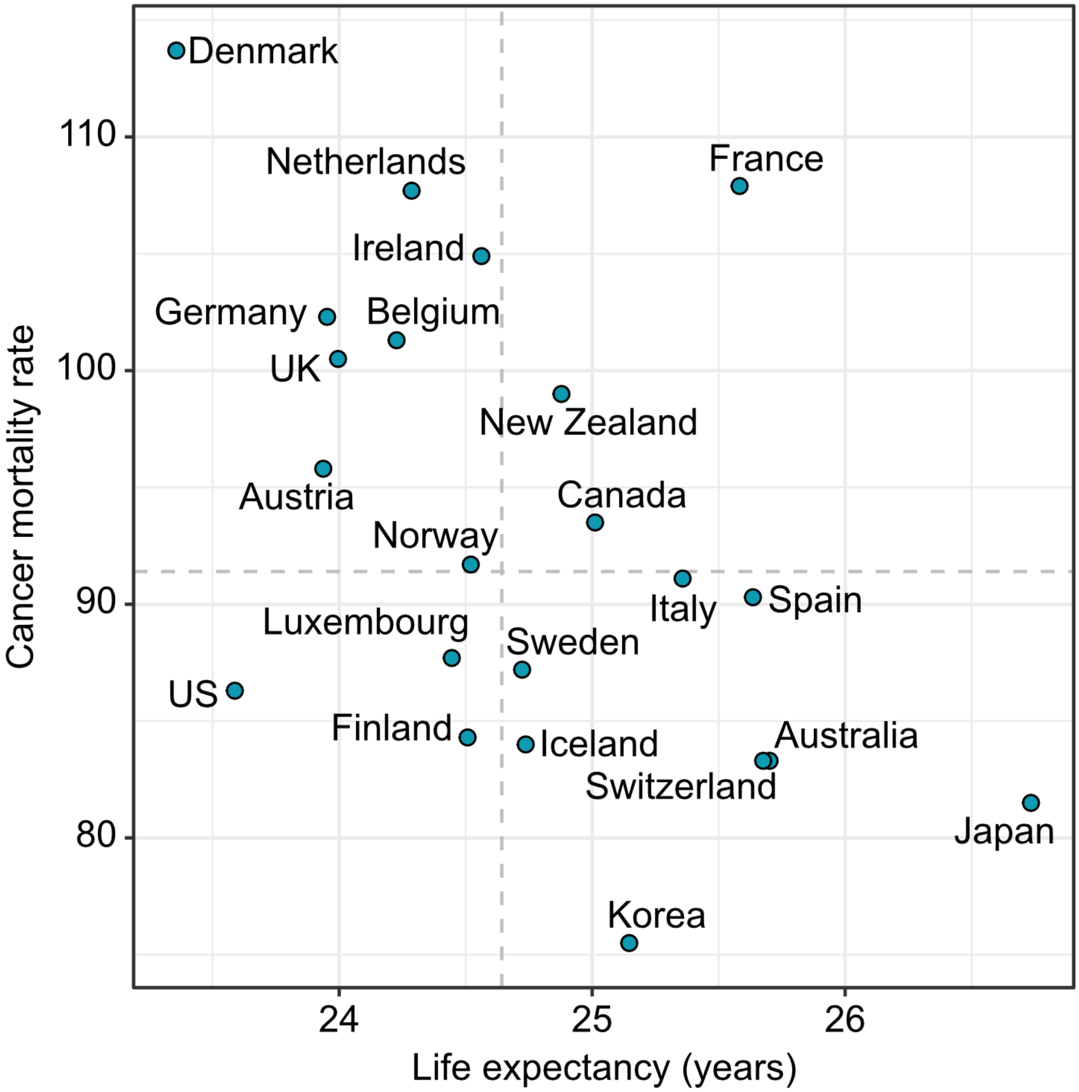

Life expectancy at age 60 in relation to age-standardized cancer mortality rates (deaths per 100,000 standard population). Dashed lines indicate the median.

© 2022 Chow RD et al. *JAMA Health Forum*.

eFigure 2: Incremental spending per cancer death averted compared with the highest-mortality country (Denmark).

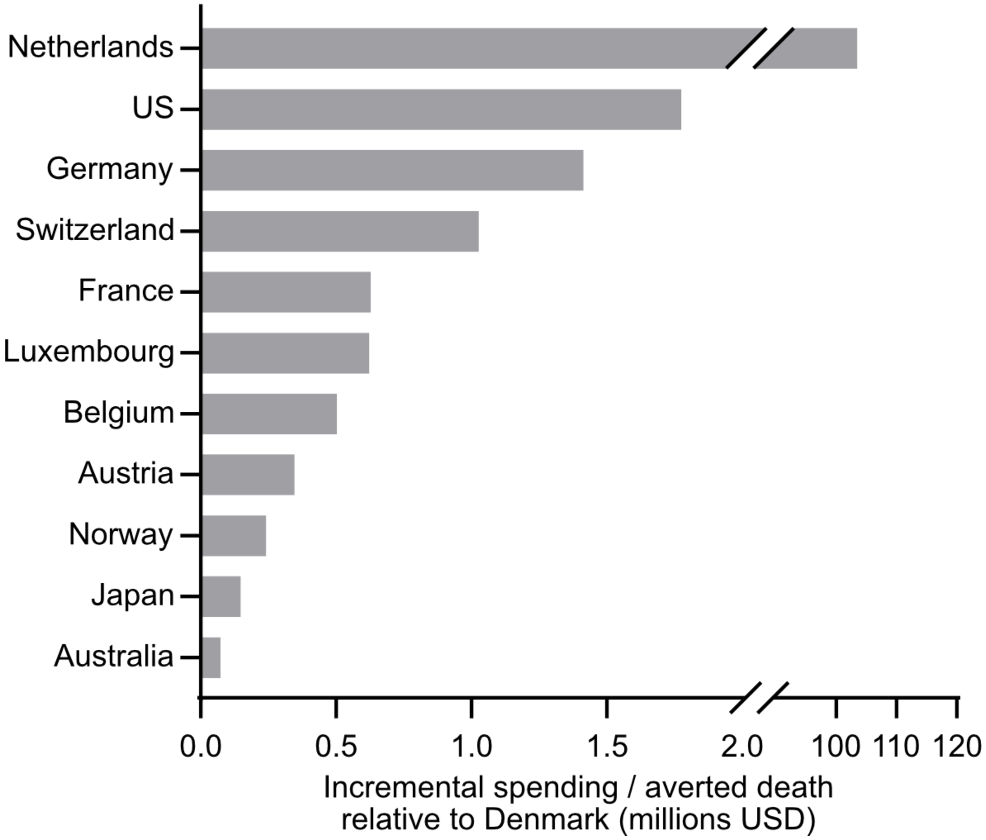

Incremental spending per cancer death averted, based on age-standardized mortality relative to Denmark. Countries with lower mortality and lower spending than Denmark were excluded from the analysis. Data are expressed in millions USD from the perspective of each country on the left.

© 2022 Chow RD et al. *JAMA Health Forum*.
